# Pixel-level bruise area quantification in strawberries using dual-band hyperspectral imaging and Efficient1DNet: Toward real-time quality monitoring

**DOI:** 10.1016/j.fochx.2026.103881

**Published:** 2026-04-17

**Authors:** Miaomiao Du, Jingyuan Zhao, Mengyao Wang, Ye Song, Xiaodong Zheng, Kang Tu, Weijie Lan, Qi Wang, Leiqing Pan

**Affiliations:** a College of Food Science and Technology, Nanjing Agricultural University, Nanjing 211800, China; b Sanya Institute of Nanjing Agricultural University, Sanya, Hainan, 572024, China; c Jinan Fruit Research Institute, All-China Federation of Supply and Marketing Cooperatives, Jinan 250220, China; d China Technology and Research Center for Storage and Processing of Fruit and Vegetables, Jinan 250220, China; e School of Life Sciences, Zhengzhou University, Zhengzhou 450001, China

**Keywords:** Strawberry, Hyperspectral imaging, Early bruise identification, Postharvest sorting, Efficient1DNet

## Abstract

High-throughput and accurate detection of early-stage bruising in strawberries is essential for online quality monitoring. This study proposes an Efficient1DNet model using visible-near infrared (Vis-NIR) and short-wave infrared (SWIR) dual-band hyperspectral imaging (HSI) to discriminate bruises and visualize spatial bruise distribution. The Vis-NIR-Efficient1DNet model provided higher classification accuracy (98.41%) for different bruised strawberries than the SWIR-Efficient1DNet model (87.83%) at post-harvest. The optimal Vis-NIR-Efficient1DNet model was applied for pixel-level bruise visualization and bruised-area quantification. For early bruised strawberries with a damaged area of 7.18 ± 2.21%, the pixel-wise calculated values showed a mean absolute error (MAE) of 1.20% and a root mean squared error (RMSE) of 1.48%. Higher detection accuracy was achieved for bruised area ratios from 19.00% to 28.90%, with MAE of 0.70% and RMSE of 0.88%. These results demonstrate that dual-band HSI combined with Efficient1DNet enables rapid, non-destructive, and scalable bruise detection for real-time quality monitoring of fresh produce.

## 1 Introduction

Strawberry, a globally important non-climacteric berry crop, is widely cultivated and consumed owing to its distinctive flavor profile and rich nutritional composition (Liu et al., 2020). The cultivar ‘Hongyan’ has gained broad recognition across China for its delicate texture, vibrant red coloration, and elevated nutritive value (Yang et al., 2024). However, strawberries are highly metabolically active and consequently extremely vulnerable to postharvest mechanical damage, among which bruising is the most prevalent form (Hussein et al., 2020). Bruising refers to the physical damage to the local tissue of a strawberry that occurs during or after harvest due to external mechanical forces (such as impacts with rigid surfaces or compression between fruits) (Li & Thomas, 2014). This mechanical stress triggers enzymatic activation, notably of cell-wall-degrading enzymes and polyphenol oxidases, thereby accelerating moisture loss, tissue softening and enzymatic browning (Lufu et al., 2020; Ma et al., 2024). Moreover, bruising compromises epidermal integrity, increasing susceptibility to microbial invasion and decay, which substantially shortens shelf life and results in considerable economic losses (Li et al., 2025). To meet modern market and distribution demands, rapid and accurate quality detection of strawberries is essential. Conventional visual inspection is labor-intensive, subjective, and inefficient; while destructive analytical methods using physical and chemical indicators, such as gas/liquid chromatography, texture puncture experiments, and microbial detection, have limitations such as strong destructiveness and inability to monitor in real time (Chang & Brecht, 2020; Satitmunnaithum et al., 2022). Consequently, the development of rapid, non-destructive, and high-precision detection techniques for identifying early-stage bruising in strawberries is of paramount importance.

Hyperspectral imaging (HSI), an emerging technology that integrates spectroscopic measurement and imaging analysis, has been extensively applied to fruit damage assessment (Mei & Li, 2023). With the rapid advancement of artificial intelligence, combining HSI with machine learning or deep learning models has greatly enhanced the accuracy, efficiency, and automation of fruit-damage detection. Prior studies have achieved successful classification of damage severity in fruits such as pears, cherries, and plums (Castillo-Girones et al., 2024; Li et al., 2024; Shao et al., 2019), and have further realized temporal prediction of bruise progression in apples, Lingwu long jujubes, blueberries, and oranges (Fan et al., 2017; Nturambirwe et al., 2021; Pourdarbani et al., 2023; Wan et al., 2025). For strawberries, Liu et al. (2018) integrated image-processing techniques with spectral feature analysis to detect both bruised and fungal-infected regions, attaining a support vector machine (SVM) accuracy of 92.59%. The SSC-AE approach effectively identified bruising, fungal infection, and soil contamination through a jointly optimized autoencoder-classifier architecture (Liu et al., 2022). Shanthini et al. (2025) employed Vis/NIR-HSI to monitor bruising across multiple postharvest stages, achieving 99.99% classification accuracy using SVM and LDA models. Feng et al. (2025) combined VNIR-HSI with a CNN-BiLSTM model, attaining 97.8% detection accuracy for slight bruising 12 h post-impact, while also enabling visual localization of bruise regions. Nevertheless, few studies have compared the discriminatory performance of Vis-NIR and SWIR dual-band HSI in distinguishing early bruised strawberries, and further explored the use of HSI technology to detect the proportion of strawberry bruise areas at different degrees of bruising.

EfficientNet, an advanced convolutional neural network (CNN) architecture, achieves an optimal balance between computational efficiency and representational power by uniformly scaling network depth, width, and input resolution (Zhang, Lu, et al., 2024). Within HSI-based applications, EfficientNet-derived architectures have demonstrated superior performance in fruit-disease detection and maturity prediction (Tan et al., 2024). However, up to now, no studies have investigated the use of EfficientNet-HSI model for bruise detection in fruits, and its application to strawberry bruise analysis remains unexplored.

**Fig. 1:**
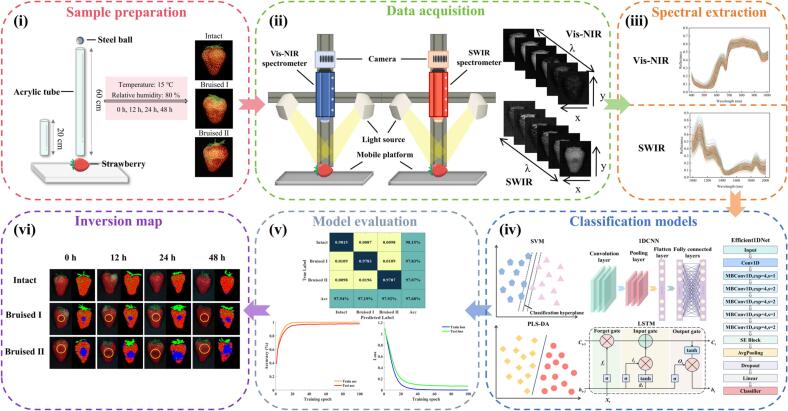
Graphical abstract (i: sample preparation; ii: data acquisition; iii: spectral extraction; iv: classification models; v: model evaluation; vi: inversion map).

In this study, we propose a dual-band HSI strategy integrated with an Efficient1DNet deep learning model for early bruise detection and spatial visualization of bruised areas in postharvest strawberries (Fig. 1). The objectives of this research are as follows:

(1) To assess the performance and feasibility of Vis-NIR and SWIR hyperspectral datasets for bruise discrimination in ‘Hongyan’ strawberries;

(2) To develop an Efficient1DNet-based deep neural network capable of rapid, accurate, and non-destructive classification of bruised samples;

(3) To validate the model's robustness and generalization during short-term storage (≤ 48 h), by quantifying bruised-area ratios through pixel-level hyperspectral visualization, thereby providing technical foundations for intelligent logistics sorting, shelf-life prediction, and supply-chain optimization in postharvest strawberry management.

## 2 Materials and methods

### 2.1 Strawberry samples and bruise experiment

Three batches of 80% mature ‘Hongyan’ strawberries free from visible mechanical damage and exhibiting uniform morphology and mass (23.48 ± 1.55 g) were collected from Tianmimi Strawberry Orchard (31.9030° N, 118.6798° E, Nanjing, China) in February, March, and April 2025. A total of 1620 samples were selected for subsequent analysis. After harvest, all strawberries were individually numbered and maintained at ambient temperature prior to experimental processing.

Bruising was induced following a modified protocol from Zhang, Liu, et al. (2024). A steel ball (20 mm in diameter, 32.6 g in weight) was released freely from heights of 0 m, 0.2 m, and 0.6 m through an acrylic tube, striking the equatorial region of each fruit to generate different impact intensities. The corresponding impact energy was calculated using Eq. (1):

(1) $$E_{i}=\mathrm{mgh}$$

where *m* is the mass of the steel ball; *g* is the gravitational constant, and *h* is the drop height of the steel ball. The resultant energies were approximately 0 J, 0.06 J and 0.19 J, corresponding to the control group (Intact), minor bruising group (Bruised I) and severe bruising group (Bruised II), respectively. All samples were equally divided into three categories (*n* = 540 per group) and stored in a constant temperature and humidity chamber (15 ± 1 °C, 80 ± 5% relative humidity) to simulate the actual post-harvest conditions of strawberries. The bruise categories (Intact, Bruised I, and Bruised II) were defined based on controlled impact conditions and corresponding epidermal damage levels, representing no damage, superficial tissue disruption, and deeper structural damage, respectively. Storage-time labels (0, 12, 24, and 48 h) were used to characterize temporal injury progression. Accordingly, bruise levels were employed for classification in tasks 1 and 3, while storage time was used in task 2, and together with the injury grade, they form the input label system of the model in the early detection task.

### 2.2 Hyperspectral image acquisition and processing

Hyperspectral images were collected using both Vis-NIR and SWIR imaging systems housed within a light-isolated chamber to minimize ambient interference. Prior to acquisition, both systems were preheated for 30 min to stabilize illumination and eliminate thermal noise. The optimal working distance between the hyperspectral lens and the fruit surface was empirically determined, and parameters were optimized for signal clarity and spectral fidelity. The Vis-NIR-HSI system employed an ImSpectorV10E imaging spectrometer (Specim, Finland; spectral range: 380–1010 nm), configured with a lens-to-sample distance of 280 mm, spectral resolution of 1.43 nm, scan rate of 6.5 mm/s, exposure time of 4 ms, and an image dimension of 804 × 1097 pixels. The SWIR-HSI system utilized a SPECIM SWIR spectrometer (Specim, Finland; 1000–2500 nm) with a working distance of 410 mm, spectral resolution of 6.20 nm, scan rate of 21 mm/s, exposure time of 1.5 ms, and an image size of 320 × 520 pixels. Hyperspectral images were acquired for three bruising groups across four storage time points (0 h, 12 h, 24 h, and 48 h). For each time point, 135 samples were analyzed per group, yielding a comprehensive dataset of 1620 hyperspectral images. Under identical optical settings, white reference images (99.99% reflectance PTFE board) and dark-current references were captured to correct for illumination variability and sensor noise, following Eq. (2):

(2) $$R_{\mathrm{Cal}}=\left(R_{\mathrm{Raw}}-R_{D}\right)/\left(R_{W}-R_{D}\right)$$

where *R*_*Cal*_ is the corrected signal intensity, *R*_*Raw*_ is the raw signal intensity, and *R*_*W*_ and *R*_*D*_ are the white and dark calibration signal intensity, respectively.

Subsequent data preprocessing involved binary masking and threshold segmentation to exclude non-fruit regions such as leaves and peduncles. The remaining fruit tissue was defined as the region of interest (ROI), from which average spectral signatures were extracted by averaging all pixel spectra within the ROI. To enhance the signal-to-noise ratio (SNR), irrelevant or noisy spectral bands were discarded, retaining the valid wavelength ranges (Vis-NIR: 380–1010 nm, 440 bands; SWIR: 1000–2000 nm, 146 bands). The spectra were smoothed using a 5-point moving average filter, followed by a first-derivative transformation to correct for baseline drift prior to modeling. For model construction, the Kennard–Stone (K—S) algorithm (Galvão et al., 2005) was used to divide the dataset into calibration and validation sets at a ratio of 2:1 for three classification tasks: (1) Severity classification of bruises (Intact, Bruised I, and Bruised II): at each time point, 267 samples were used for calibration and 138 for validation; (2) Classification of storage time (0 h, 12 h, 24 h, and 48 h) within each bruise group: 356 and 184 samples were used for calibration and validation; (3) Early detection of all bruise grades (≤ 48 h): 1068 samples were used for calibration and 552 for validation.

### 2.3 Determination of *L*^*⁎*^, *a*^*⁎*^, *b*^*⁎*^ values, bruised area, firmness, SSC and TA

The surface color of bruised and intact regions was measured using a CR-400 colorimeter (Konica Minolta, Tokyo, Japan), recording *L*^*⁎*^, *a*^*⁎*^, *b*^*⁎*^ values in the CIELAB color space.

The bruised area (*S*) (Mohsenin et al., 1978) was calculated according to Eq. (3):

(3) S=πab

where *a* and *b* represent the semi-major and semi-minor axes of the elliptical bruise region, respectively. The projected top-view area of the strawberry is approximately an ellipse. Its area is calculated based on the semi-major and semi-minor axes of the projection contour, and the calculation formula is similar to the above equation.

Fruit firmness was evaluated with a TMS-Pro texture analyzer (FTC, USA) under standardized conditions: initial force 0.2 N, penetration speed 60 mm/min, and depth 5 mm.

The soluble solids content (SSC) was quantified using a digital refractometer (PAL-1, ATAGO, Tokyo), while titratable acidity (TA) was determined with an automatic titrator (PAL-Easy Acid F5, ATAGO, Tokyo) (Wang, Li, et al., 2025).

### 2.4 Classification algorithms

Five classification models were constructed for the strawberry data using support vector machine (SVM), partial least squares-discriminant analysis (PLS-DA), one-dimensional convolutional neural network (1DCNN), long short-term memory (LSTM), and Efficient1DNet. The performance and computational efficiency of these five models were evaluated.

SVM is a supervised learning algorithm grounded in the principle of structural risk minimization, employing kernel-based transformations to project input data into high-dimensional feature space, where an optimal hyperplane maximizes interclass separation (Cong et al., 2025). This enables robust generalization for small-sample, nonlinear, and high-dimensional classification problems.

PLS-DA is a multivariate discriminant approach combining the merits of principal component analysis (PCA), canonical correlation analysis (CCA), and multiple linear regression (MLR). It projects predictor-response pairs into a latent subspace that maximizes interclass variance while mitigating multicollinearity, thereby ensuring stable and interpretable classification in complex spectral datasets (Chun et al., 2024).

1DCNN is a hierarchical deep feedforward neural architecture comprising convolutional, pooling, and fully connected layers, employing local connectivity and shared weights to extract progressively abstract spectral features (Chuquimarca et al., 2024). Its end-to-end learning mode not only enables efficient modeling of high-dimensional sequential spectral data (Liu et al., 2025), but also allows for model interpretability analysis, thereby enhancing the reliability of the model (Wang et al., 2025, Wang et al., 2026).

The LSTM network architecture is fundamentally composed of three gating components: the forget gate, input gate, and output gate. Through this distinctive gating mechanism integrated with memory cells, the LSTM effectively mitigates the gradient vanishing and explosion problems that commonly afflict conventional recurrent neural networks (RNNs) when processing long sequential data, making LSTM a dominant architecture for time-series and sequence modeling tasks (Zhong et al., 2023).

EfficientNet, originally proposed by Tan and Le (2020), is an advanced lightweight CNN architecture that combines neural architecture search (NAS) with an optimized compound scaling strategy. Its core innovation is a coordinated compound scaling strategy that jointly adjusts depth, width, and resolution. The model employs a unified compound scaling coefficient φ to perform polynomial scaling ($\alpha^{\varphi },\beta^{\varphi }$,$\gamma^{\varphi }$) across these dimensions, ensuring balanced capacity growth and computational efficiency, as formulated in Eq. (4):

$$\text{depth}:d=\alpha^{\varphi }$$

$$\text{width}:w=\beta^{\varphi }$$

(4) $$\text{resolution}:r=\gamma^{\varphi }$$

$$s.t.\alpha ∙\beta^{2}∙\gamma^{2}\approx 2$$

α≥1,β≥1,γ≥1

where *α*, *β*, and *γ* denote the baseline scaling coefficients for depth, width, and resolution, respectively, which are determined through grid-search optimization. The compound coefficient φ serves as the primary scaling parameter that governs the overall model capacity (Wang, Wei, et al., 2025). As φ increases, the three dimensions expand synchronously according to predetermined scaling ratios. Compared with traditional one-dimensional scaling strategies, EfficientNet achieves an optimal balance between computational efficiency and model representational power, resulting in enhanced accuracy with minimal resource overhead.

To adapt EfficientNet for one-dimensional hyperspectral data, we developed a lightweight Efficient1DNet architecture. The model retains the core architectural modules of EfficientNet, namely the mobile inverted bottleneck convolution (MBConv) and squeeze-and-excitation (SE) blocks, while transforming them into 1D convolutional operations. The model processes an input sequence $x\in {\mathbb{R}}^{B\times L}$ by first expanding its channel dimension to $\left[B,1,L\right]$, followed by a sequence of convolutional encoding, MBConv blocks augmented with SE attention, and global pooling for final classification. The complete process is mathematically expressed in Eqs. (5), (6), (7), (8):

(5) $$X_{1}=\text{SiLU}\left(\mathrm{BN}\left(\text{Conv}1D_{1\to C_{0}}^{\left(k_{0},s_{0},p_{0}\right)}\left(x\right)\right)\right)$$

Given an input $U\in {\mathbb{R}}^{B\times C\times L}$, with expansion factor e≥1 and $\hat{C}=\mathrm{eC}$, the MBConv block performs the following operations:

$$H_{1}=\text{SiLU}\left(\mathrm{BN}\left({\mathrm{PW}}^{C\to \hat{C}}\left(U\right)\right)\right)$$

$$H_{2}=\text{SiLU}\left(\mathrm{BN}\left({\mathrm{DW}}^{k,s,p}\left(H_{1}\right)\right)\right)$$

(6) $$a=\sigma \left(W_{2}\text{ReLU}\left(W_{1}\mathrm{GAP}\left(H_{2}\right)\right)\right)$$

$$V=\mathrm{BN}\left({\mathrm{PW}}^{\hat{C}\to C^{'}}\left(H_{2}⊙a\right)\right)$$

A residual connection is optionally applied as:

(7) $$Y=\left\{\begin{array}{c} U+V,\text{if}\;s=1\;\text{and}\;C^{\prime }=C\\ V,\text{otherwise} \end{array}\right)$$

Specifically, within the MBConv block, the expansion convolution, depthwise convolution, and projection convolution can respectively strengthen the subtle differences between adjacent wavelengths, extract local continuous spectral patterns, and suppress redundant features. This design is highly consistent with the intrinsic characteristics of spectral signals, which exhibit local smoothness and sparsely distributed key absorption peaks. Meanwhile, the channel attention mechanism provided by SEBlock can adaptively adjust the importance of different bands, enabling the model to pay more attention to the absorption regions related to damage changes and reducing the interference of background noise. Moreover, the lightweight 1D convolution framework significantly reduces the parameter size while maintaining the expression ability, making it more suitable for scenarios with relatively limited spectral sample quantities.

After multiple MBConv layers, the final feature **F** is globally pooled and passed through a fully connected classifier:

$$g=\mathrm{GAP}\left(F\right)$$

(8) $$\hat{y}=\text{Softmax}\left(W_{g}+b\right)$$

Here, $x\in {\mathbb{R}}^{B\times L}$ denotes the input spectral sequence, and $X_{1}\in B\times C_{0}\times L_{1}$ represents the stem convolution output. In MBConv, U and Y are the input and output with channels C*,*$C^{\prime }$, and expanded $\hat{C}=\mathrm{eC}$. The depthwise convolution uses kernel size k, stride s, and padding p, and $W_{1}$, $W_{2}$ are the SE weights. The excitation vector a regulates the channel-wise attention, g represents the globally pooled feature, and $\hat{y}$ corresponds to the final classification output. Other notations include: BN for batch normalization, PW for pointwise convolution, DW for depthwise convolution, GAP for global average pooling, $\text{SiLU}\left(z\right)=z∙\sigma \left(z\right)$ as activation, and ⊙ for element-wise multiplication. The training of the Efficient1DNet model uses the Adam optimizer with a learning rate of 5 × 10^−5^, and uses the CrossEntropyLoss function for multi-class training. The training data is batched loading with a batch size of 32, and the training set is randomly shuffled (shuffle = True), while the validation set also uses a batch size of 32. Each round of training lasts for 100 epochs. To ensure the stability and reproducibility of the model, we conducted 5 independent repeated training and recorded the classification accuracy of the training set and validation set for each training. Finally, among the five repeated experiments, the run with the highest validation accuracy was selected, and the corresponding parameter settings were saved.

### 2.5 Model evaluation and statistical analysis

This study systematically assessed the performance and generalization ability of the classification models using standard evaluation metrics, including overall accuracy, confusion matrices of the validation sets, and training-validation accuracy and loss convergence curves. To quantify the reliability of the classification model in identifying the bruised area, the mean absolute error (MAE) and root mean squared error (RMSE) were employed. These metrics assess the deviation between the pixel-wise proportion of the bruised area in the hyperspectral image and the measured values, thereby reflecting the consistency between the hyperspectral inversion results and the real situation, as formulated in Eqs. (9), (10), (11):

(9) $$\text{Accuracy}=\frac{\mathrm{TP}+\mathrm{TN}}{\mathrm{TP}+\mathrm{FP}+\mathrm{TN}+\mathrm{FN}}$$

(10) $$\mathrm{MAE}=\frac{1}{n}\sum_{i=1}^{n}\left|y_{\mathrm{true},i}-y_{\mathrm{pred},i}\right|$$

(11) $$\text{RMSE}=\sqrt{\frac{1}{n}\sum_{i=1}^{n}{\left(y_{\mathrm{true},i}-y_{\mathrm{pred},i}\right)}^{2}}$$

FP, TP, FN, and TN represent the counts of false positives, true positives, false negatives, and true negatives; and $y_{\mathrm{true},i}$ and $y_{\mathrm{pred},i}$ are the true and predicted values of the *i*-th sample, respectively.

Hyperspectral data preprocessing and feature extraction were performed in MATLAB R2024a (MathWorks Inc., Natick, MA, USA), whereas all deep learning architectures were implemented and trained using PyTorch 2.0.0 (Meta Platforms Inc., Menlo Park, CA, USA) within the Python 3.12.3 environment (Python Software Foundation, Wilmington, DE, USA).

All experiments were conducted in triplicate, and the resulting data were expressed as mean ± standard deviation (SD). The single-factor analysis of variance (ANOVA) for the physicochemical indicators was conducted using IBM SPSS 27 (SPSS Inc., Chicago, IL, USA), and the Duncan test was performed at *P < 0.05*. Charts for all quality parameters were drawn using Origin 2024 (OriginLab Corp., Northampton, MA, USA).

## 3 Results and discussion

### 3.1 Physicochemical changes in bruised strawberries

**Fig. 2:**
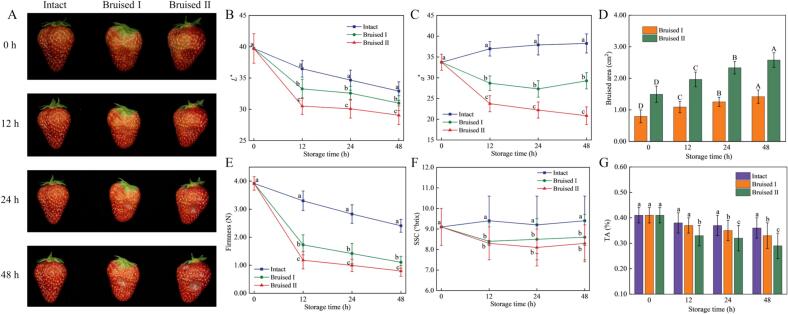
The physicochemical evolution of ‘Hongyan’ strawberries with different bruising levels during 2 days of storage (A: appearance comparison; B: *L*^*⁎*^ (lightness); C: *a*^*⁎*^ (redness); D: bruised area; E: firmness; F: SSC; G: TA).

The physicochemical evolution of ‘Hongyan’ strawberries with different bruising levels during 2 days of storage is illustrated in Fig. 2. The Bruised II group showed visible softening and discoloration at 0 h, whereas the Bruised I samples showed less distinct surface deformation (Fig. 2A). The color parameters (*L*^*⁎*^*, a*^*⁎*^*, b*^*⁎*^) of bruised fruits differed significantly (*P*  <  0.05) from those of intact samples throughout 2 days of storage (Fig. 2B, C**; Fig. S1**). The Bruised II samples displayed the lowest brightness and chromatic values at 48 h. Mechanical bruising disrupts cellular integrity, facilitating the interaction of intracellular enzymes and phenolic substrates, thereby inducing oxidative browning, pigment degradation, and metabolic imbalance (Hussein et al., 2019). As storage time progressed, the bruised regions expanded markedly (Fig. 2D). The bruised area of the Bruised I group increased from the initial 0.79 cm^2^ to 1.42 cm^2^, while that of the Bruised II group expanded significantly from 1.50 cm^2^ to 2.58 cm^2^. Firmness decreased in all groups over time and the rate of softening was substantially higher in bruised samples (Fig. 2E). This indicates that greater impact energy results in more extensive structural collapse and thus accelerated tissue softening.

The SSC of the Intact group remained relatively stable throughout storage (Fig. 2F). In contrast, bruised samples (Bruised I/II) exhibited a progressive decline in SSC, with values significantly lower than those of intact fruits. This decline suggests that bruise-induced respiration accelerates sugar catabolism, thereby depleting soluble solids (Xie et al., 2021). During the early storage stage (12*h*), TA of the Bruised I group did not differ significantly (*P > 0.05*) from that of the Intact group (Fig. 2G). However, after 24–48 h, TA values in bruised strawberries decreased sharply, becoming substantially lower than those of intact fruits, indicating that mechanical injury accelerates the breakdown of organic acids. Unlike physical indicators (e.g., firmness), chemical indices (SSC and TA) showed stronger time dependence due to ongoing biochemical deterioration. Physical indicators reflect cell integrity and mechanical damage, changing faster in Bruised I/II samples from impact-induced tissue collapse. Chemical indicators reflect metabolic and enzymatic activities, with their time-dependent changes driven by sugar degradation, acid decomposition, and oxidation.

### 3.2 Spectral characterization of bruised strawberries

**Fig. 3:**
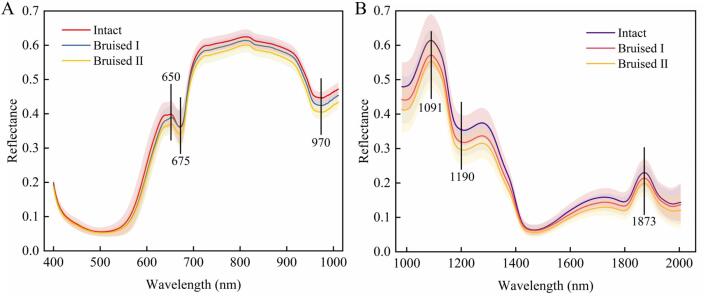
Average Vis-NIR (A) and SWIR (B) reflectance curves of strawberries with different bruising levels during 2 days of storage.

As shown in Fig. 3, the reflectance of bruised samples was consistently lower than that of intact fruits in both spectral ranges, particularly within the 420–650 nm and 710–1000 nm intervals. The distinct reflectance changes near 450 nm and 650 nm correspond to the oxidative breakdown of chlorophyll, anthocyanins, and carotenoids, respectively (Pan et al., 2016). The enhanced absorption valley around 675 nm reflects increased pigment degradation (Gao et al., 2020; Weng et al., 2020). The 710–1000 nm region is highly sensitive to water absorption and internal structural changes. Bruising causes the free water in the strawberry tissue to seep out. At around 970 nm, the in-phase stretching vibration of the O—H bond in water molecules leads to the appearance of a strong absorption peak (Nagata et al., 2006).

In the SWIR spectral region, the 1000–1300 nm region is mainly the secondary doublet absorption zone of the O—H bonds in water molecules (Keresztes et al., 2017). Near 1191 nm, bruised samples exhibit significantly reduced reflectance relative to intact fruits, a phenomenon likely caused by tissue disruption that accelerates intracellular water migration and redistribution (Keresztes et al., 2016). Spectral differences in the 1600–2000 nm range can be ascribed to the combination bands of C—H and O—H stretching vibrations in sugars and other organic compounds (Li et al., 2022). A strong absorption band near 1873 nm further highlights the dominant influence of water content in bruised tissues. These changes reflect the progressive deterioration in bruised strawberries, including cell membrane rupture, intracellular water migration, and tissue disorganization.

### 3.3 Classification models for bruised strawberries

#### 3.3.1 Graded identification of different bruising levels

Classification models for different degrees of bruising of strawberries during 2 days of storage based on Vis-NIR/SWIR-HSI are shown in Table 1, Table 2. Results demonstrated that the performance of models in the Vis-NIR band is generally superior to that in the SWIR band. This difference stems from the higher sensitivity of the Vis-NIR range (550–750 nm) to early pigment degradation and slight structural damage, whereas the SWIR region (1000–2000 nm), dominated by water and organic absorption and affected by lower spatial resolution and higher noise, is less responsive to subtle bruising but more informative at later stages of tissue degradation. The Vis-NIR-Efficient1DNet model achieved the best overall performance, attaining 100.00% calibration accuracy and maintaining a validation accuracy exceeding 98.00%. This result surpasses the highest accuracy (97.8%) reported in Feng et al. (2025) for strawberry bruise classification. Both 1DCNN and LSTM models exhibited comparable yet slightly lower accuracies. The conventional SVM and PLS-DA algorithms displayed relatively lower discrimination at 0 h. Within the SWIR spectral region, even the top-performing Efficient1DNet model achieved only 87.83% validation accuracy at 0 h. The SVM model showed relatively stable performance throughout 12–48 h. However, the highest validation set accuracy of the PLS-DA model was only 91.30%. Both 1DCNN and LSTM models showed notable performance fluctuations. During the early postharvest stage (0 h), SVM, PLS-DA, 1DCNN, and LSTM models performed suboptimally, while the Vis-NIR-Efficient1DNet model maintained remarkable robustness, achieving 98.41% validation accuracy. This superior performance is attributed to the compound scaling strategy embedded within Efficient1DNet, which jointly optimizes network depth, width, and convolutional kernel resolution. Such design facilitates efficient multi-scale feature extraction, enabling the model to capture subtle spectral differences related to incipient bruising (Huang et al., 2024).

**Table 1:** Classification models for different degrees of bruising of strawberries during 2 days of storage based on Vis-NIR-HSI.

Models	Storage time	**Calibration set (%)**					**Validation set (%)**			
		Intact	Bruised I	Bruised II	Overall		Intact	Bruised I	Bruised II	Overall
SVM	0 h	91.01	78.65	98.88	89.51		93.48	73.91	97.83	88.41
	12 h	97.75	98.88	97.75	98.13		95.65	100.00	97.83	97.83
	24 h	98.88	86.52	100.00	95.13		100.00	84.78	97.83	94.20
	48 h	97.75	93.26	97.75	96.25		95.65	91.30	100.00	95.65
PLS-DA	0 h	93.26	88.76	77.53	86.52		95.65	82.61	76.09	84.78
	12 h	95.51	100.00	98.88	98.13		97.83	95.65	100.00	97.83
	24 h	94.38	97.75	94.38	95.51		93.48	97.83	91.30	94.20
	48 h	100.00	98.88	100.00	99.63		97.83	100.00	100.00	99.28
1DCNN	0 h	95.96	94.94	93.60	94.83		93.83	93.04	91.91	92.93
	12 h	100.00	93.48	90.34	94.61		100.00	90.87	86.96	92.61
	24 h	96.85	90.00	99.55	95.47		94.13	89.00	97.83	93.65
	48 h	88.31	98.88	90.56	92.58		87.83	97.96	89.57	91.79
LSTM	0 h	99.10	100.00	100.00	99.70		88.26	93.91	89.83	90.67
	12 h	95.96	91.01	98.20	95.06		90.87	88.74	92.61	90.74
	24 h	97.98	95.51	95.51	96.33		92.61	89.57	88.26	90.14
	48 h	95.28	97.75	98.88	97.30		93.91	86.09	95.22	91.74
**Efficient1DNet**	**0 h**	**100.00**	**100.00**	**100.00**	**100.00**		**99.13**	**98.26**	**97.83**	**98.41**
	**12 h**	**100.00**	**100.00**	**100.00**	**100.00**		**99.57**	**99.13**	**99.57**	**99.42**
	**24 h**	**100.00**	**100.00**	**100.00**	**100.00**		**99.57**	**99.57**	**100.00**	**99.71**
	**48 h**	**100.00**	**100.00**	**100.00**	**100.00**		**100.00**	**99.57**	**100.00**	**99.86**

**Table 2:** Classification models for different degrees of bruising of strawberries during 2 days of storage based on SWIR-HSI.

Models	Storage time	**Calibration set (%)**					**Validation set (%)**			
		Intact	Bruised I	Bruised II	Overall		Intact	Bruised I	Bruised II	Overall
SVM	0 h	96.63	87.64	87.64	90.64		100.00	82.61	82.61	88.41
	12 h	96.63	95.51	97.75	96.63		95.65	91.30	91.30	92.75
	24 h	97.75	95.51	93.26	95.51		100.00	95.65	86.96	94.20
	48 h	97.75	92.13	97.75	95.88		97.83	93.48	91.30	94.20
PLS-DA	0 h	87.64	82.02	92.13	87.27		91.30	84.78	82.61	86.23
	12 h	92.13	85.39	89.89	89.14		89.13	84.78	86.96	86.96
	24 h	88.76	88.76	91.01	89.51		84.78	100.00	82.61	89.13
	48 h	95.51	85.39	97.75	92.88		93.48	84.78	95.65	91.30
1DCNN	0 h	82.92	89.78	84.49	85.73		82.61	87.39	82.87	84.29
	12 h	90.00	88.09	94.61	90.90		87.83	86.13	92.43	88.79
	24 h	93.26	88.54	94.38	92.06		93.04	84.78	92.17	89.99
	48 h	92.81	94.61	97.53	94.98		97.39	91.74	94.26	94.46
LSTM	0 h	91.24	85.17	96.85	91.09		88.30	83.36	92.91	88.19
	12 h	94.83	95.39	90.00	93.41		90.39	92.22	86.91	89.84
	24 h	97.75	97.53	99.33	98.20		91.30	93.13	97.83	94.09
	48 h	96.85	91.35	98.89	95.69		94.00	89.52	96.78	93.43
**Efficient1DNet**	**0 h**	**100.00**	**100.00**	**100.00**	**100.00**		**89.57**	**84.78**	**89.13**	**87.83**
	**12 h**	**100.00**	**100.00**	**100.00**	**100.00**		**96.52**	**91.74**	**92.17**	**93.48**
	**24 h**	**100.00**	**100.00**	**100.00**	**100.00**		**95.65**	**96.52**	**99.57**	**97.25**
	**48 h**	**100.00**	**100.00**	**100.00**	**100.00**		**98.70**	**95.22**	**94.35**	**96.09**

**Fig. 4:**
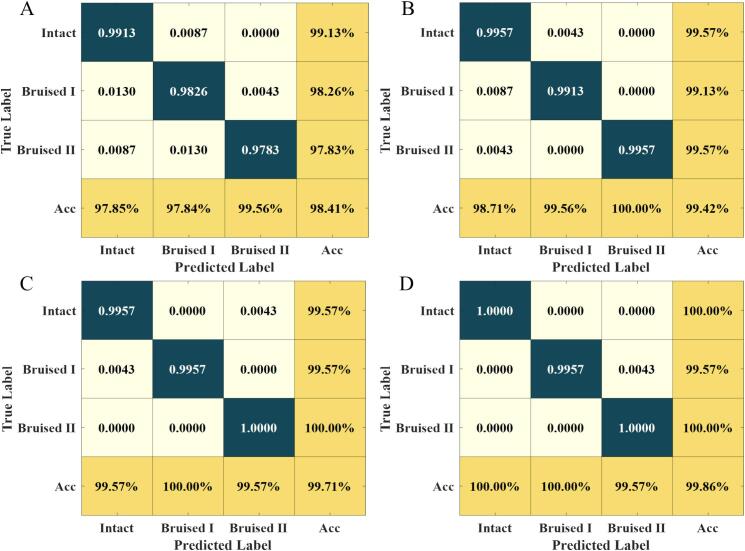
Validation set confusion matrices for strawberry classification across four storage intervals using the optimal Vis-NIR-Efficient1DNet model (A: 0 h; B: 12 h; C: 24 h; D: 48 h).

The confusion matrices for strawberry classification across four storage intervals (0 h, 12 h, 24 h, and 48 h) using the optimal Vis-NIR-Efficient1DNet model are shown in Fig. 4. The Bruised I category was relatively more difficult to distinguish compared with the Intact and Bruised II groups, yielding classification accuracies of 98.26% and 99.13% at 0 h and 12 h, respectively (Figs. 4A, B). Bruised I samples were occasionally misclassified as Intact (1.30%), whereas Bruised II samples were sometimes identified as Bruised I (1.30%) at 0 h. Spectral differences between adjacent bruise levels are mainly reflected in subtle variations of local absorption features, leading to partial spectral overlap at early stages. In addition, the gradual accumulation of spectral differences over storage time and the relatively similar sample distributions further increase the likelihood of misclassification for a small number of samples. Bruise progression affected classification difficulty, underscoring the importance of time-aware spectral modeling.

#### 3.3.2 Classification of storage time with identical bruise levels

The storage-time classification models of strawberries with identical bruise levels based on Vis-NIR/SWIR-HSI are presented in **Tables S1 and S2**. All constructed models demonstrated their feasibility, with the Vis-NIR-Efficient1DNet model achieving the highest accuracy. Validation accuracies reached 99.24%, 96.20%, and 98.48% for Intact, Bruised I, and Bruised II groups, respectively. The confusion matrices show that samples from adjacent time points with identical bruise levels demonstrate higher misclassification rates. The 0 h Bruised I samples show 2.17% and 4.35% misclassification rates as 12 h and 24 h samples respectively (**Fig. S5B**). Similarly, 0 h Bruised II samples exhibit 1.74% and 3.04% misclassification rates to 12 h and 24 h (**Fig. S5C**). This indicates that strawberry quality changes relatively slowly within the initial 24 h after bruising, with minimal spectral characteristic differences (Yuan et al., 2022). However, from 24 to 48 h, as the quality changes became more pronounced, the accuracy of storage-time classification also increased.

#### 3.3.3 Classification of bruised strawberries across all storage periods

As summarized in Table 3, compared with the bruise classification model based on a single storage time point, the model performance decreases slightly when all storage time points are incorporated. Nevertheless, the Efficient1DNet model retained superior generalization capability, achieving validation accuracies of 97.68% (Vis-NIR) and 97.38% (SWIR), highlighting its robust cross-temporal learning capacity. The confusion matrix of the optimal Vis-NIR-Efficient1DNet model (**Fig. S5D**) indicates that Bruised II samples were most frequently misclassified as Bruised I (1.96%). The accuracy and loss curves of the Vis-NIR-Efficient1DNet model across all three classifications tasks (3.3.1, 3.3.2, 3.3.3) are shown in **Fig. S6**, demonstrating rapid and stable convergence with minimal overfitting. The high degree of agreement between training and validation losses confirms the model's robustness, computational efficiency, and scalability for real-world implementation in non-destructive bruise detection systems.

**Table 3:** Classification models based on Vis-NIR/SWIR-HSI for different degrees of bruising of strawberries during 2 days of storage (0–48 h).

Spectral region	Models	**Calibration set (%)**				**Validation set (%)**			
		Intact	Bruised I	Bruised II	Overall	Intact	Bruised I	Bruised II	Overall
Vis-NIR	SVM	95.79	90.17	95.79	93.91	95.11	82.07	97.83	91.67
	PLS-DA	96.35	85.39	91.57	91.10	97.83	76.09	94.57	89.49
	1DCNN	99.49	81.29	82.75	87.85	99.13	78.26	79.89	85.76
	LSTM	100.00	99.94	99.83	99.93	91.09	86.52	89.46	89.02
	**Efficient1DNet**	**100.00**	**100.00**	**100.00**	**100.00**	**98.15**	**97.83**	**97.07**	**97.68**
SWIR	SVM	94.10	90.45	95.22	93.26	92.39	90.22	94.02	92.21
	PLS-DA	92.98	91.29	92.70	92.32	92.39	90.22	90.76	91.12
	1DCNN	82.75	86.57	89.78	86.37	88.15	85.28	84.78	86.07
	LSTM	93.54	90.79	92.56	92.30	93.15	85.43	87.72	88.77
	**Efficient1DNet**	**98.54**	**98.76**	**98.71**	**98.67**	**95.98**	**98.70**	**97.46**	**97.38**

### 3.4 Visualization of bruised area and model efficiency analysis

**Fig. 5:**
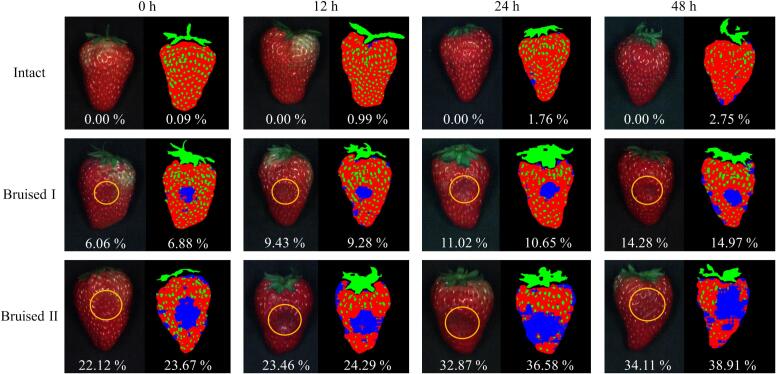
The pixel-wise bruise identification and quantitative visualization of strawberries with different bruising levels during 2 days of storage (Blue represents bruised tissue, red/orange represents intact flesh, and green represents excluded non-target regions (seeds and leaves)). (For interpretation of the references to color in this figure legend, the reader is referred to the web version of this article.)

For strawberries with different bruising levels, the ROIs encompassing the entire fruit surface were extracted using Otsu threshold segmentation combined with morphological refinement of grayscale images acquired at the characteristic wavelength of 647 nm. The selection of 647 nm was based on its strong and stable reflectance contrast between different bruise levels and storage times (640–660 nm), which helps to enhance the contrast between the target area and the background, as well as its robustness in segmentation across varying conditions, enabling accurate ROI extraction and consistent pixel-level visualization. Subsequently, achene regions were segmented via black-hat morphological transformation, while leaf fragments were identified and removed based on normalized difference vegetation index (NDVI) thresholding. The optimal Vis-NIR-Efficient1DNet model was employed to conduct pixel-level classification. The bruised-area ratio was computed as the proportion of pixels classified as damaged relative to the total pixel count of each strawberry. Fig. 5 illustrates the pixel-wise bruise identification and quantitative visualization for representative strawberry samples. Each specimen displays its original visible-light image (left) alongside the classification map (right), with yellow circles denoting the impact sites. In the color-coded classification maps, blue indicates bruised tissue, red/orange represents intact flesh, and green corresponds to excluded non-target regions (seeds and leaves). The percentages presented below each pair of images represent the actual measured and calculated bruised-area ratios.

The three representative sample groups correspond to Intact, Bruised I, and Bruised II conditions, respectively. Samples in the first row exhibited bruised-area ratios below 3%, indicating that most tissues remained structurally intact. The second row displayed ratios between 6% and 15%, characteristic of mild bruising with localized discoloration. In contrast, the third row demonstrated extensive contiguity of bruised regions exceeding 38%. The blue area formed a large contiguous area, presenting a typical severe bruising condition. This progressive spatial transition closely matched the visual appearance of the fruits, confirming the high spatial fidelity and physiological relevance of the model's pixel-level predictions. Further observation revealed that bruised pixels were predominantly concentrated around the initial impact point, forming patch-like or annular diffusion patterns indicative of internal mechanical propagation pathways.

As summarized in Table 4, the MAE of the pixel-level quantification results for intact strawberries increased from 0.34% to 2.55% over the storage period. This may be attributed to surface color changes and slight misclassification. The pixel-level quantification results of the model for Bruised I samples showed excellent agreement with the measured bruised area ratio, with the MAE decreasing continuously from 1.20% at 0 h to 0.81% at 48 h, and the RMSE dropping from 1.48% to 0.97%. For the Bruised II samples, the pixel-level bruised-area ratios were basically consistent with the measured values during the early storage period (0−12*h*) (with MAEs of 0.70% and 0.71% respectively). Although the model maintained accurate spatial recognition, the pixel-level calculated ratios tended to be slightly higher than the measured ones after 48 h (MAE increasing to 2.60%; RMSE reaching 2.89%). This suggests that the enhanced spectral sensitivity of the model at later stages, combined with tissue deterioration which produces greater variation in absorption and scattering, caused the classifier to expand bruise boundaries and slightly overestimate affected areas. Nevertheless, the Vis-NIR-Efficient1DNet model reliably captured the spatial distribution and progression of bruising. When compared with the CNN-BiLSTM-based visualization framework reported by Feng et al. (2025), the present approach provides an additional quantitative advantage by computing the bruised-area ratio directly from pixel counts, thereby enhancing its practical applicability in postharvest bruise assessment and automated grading systems.

**Table 4:** Comparison between the measured and the pixel-level calculated bruised area ratios in strawberries with different bruising levels.

**Groups**	**Indicators (%)**	**Storage time**			
		**0 h**	**12 h**	**24 h**	**48 h**
Intact	Actual values	0	0	0	0
	Pixel-level calculation	0.34 ± 0.23	1.14 ± 0.55	1.94 ± 1.01	2.55 ± 1.02
	MAE	0.34	1.14	1.94	2.55
	RMSE	0.41	1.26	2.19	2.74
Bruised I	Actual values	7.18 ± 2.21	9.68 ± 2.48	10.72 ± 3.39	15.05 ± 3.73
	Pixel-level calculation	7.78 ± 1.62	9.97 ± 2.00	11.57 ± 3.17	15.55 ± 3.51
	MAE	1.20	0.99	0.92	0.81
	RMSE	1.48	1.27	1.11	0.97
Bruised II	Actual values	23.95 ± 4.95	25.27 ± 4.85	35.00 ± 4.20	37.48 ± 5.21
	Pixel-level calculation	23.79 ± 4.77	25.57 ± 4.75	36.69 ± 4.41	39.34 ± 5.14
	MAE	0.70	0.71	1.90	2.60
	RMSE	0.88	0.92	2.18	2.89

Table 5 compares the key performance indicators, including parameter count, model size, computational efficiency, and overall accuracy, across the 1DCNN, LSTM, and Efficient1DNet architectures. The Efficient1DNet achieved 405,098 parameters, a compact model size of 1.6 MB, and a runtime of 8.62 s per run (5 runs × 100 epochs), while maintaining an overall accuracy of 97.68%. In contrast, the conventional 1DCNN, despite possessing a larger capacity (903,460 parameters; 3.45 MB), reached only 85.76% accuracy and required the longest runtime (11.91 s per run), indicating limited scalability. Although the LSTM model was lightweight (48,197 parameters, 0.19 MB) and offered the shortest runtime (7.49 s per run), its highest accuracy of 89.02%, which is significantly lower than that of Efficient1DNet.

**Table 5:** Comparison of model efficiency parameters among 1DCNN, LSTM, and Efficient1DNet for Vis-NIR-based strawberry bruise classification.

**Model**	**Parameter count**	**Model size (MB)**	**Runtime per run (s)**	**Overall accuracy (%)**
1DCNN	903,460	3.45	11.91	85.76
LSTM	48,197	0.19	7.49	89.02
Efficient1DNet	405,098	1.6	8.62	97.68

## 4 Conclusion

This study developed and validated an Efficient1DNet model based on dual-band Vis-NIR/SWIR-HSI, which was used to distinguish the ‘Hongyan’ strawberries of different bruising levels and storage times, and to visualize the spatial distribution of the bruises on the strawberries. The Vis-NIR/SWIR-Efficient1DNet outperformed SVM, PLS-DA, LSTM, and 1DCNN. Notably, the Vis-NIR-Efficient1DNet achieved 98.26% accuracy for Bruised I samples at 0 h postharvest. For storage-time classification at identical bruise levels, the optimal Vis-NIR-Efficient1DNet achieved 90.87% accuracy for Bruised I and 95.22% for Bruised II samples at 0 h. When all storage intervals (0–48 h) were integrated, validation accuracies were 97.68% (Vis-NIR) and 97.38% (SWIR). Pixel-wise inversion combined with mask-based segmentation enabled accurate bruise localization and bruise-area quantification. For early Bruised I samples (0–12 h), pixel-level bruise quantification showed low errors (MAE ≤ 1.20%, RMSE ≤1.48%).

Despite the promising performance of the proposed model, several limitations should be acknowledged. The samples were derived from a single source, with limited diversity in cultivars, maturity stages, and naturally bruised fruit, which may constrain the generalizability of the model. In addition, the simulated bruising applied in this study may not fully capture the complexity of mechanical stresses encountered under real postharvest conditions, particularly across different production regions and transportation scenarios, where spectral responses are likely to be more heterogeneous and model performance may decline. Future work will focus on expanding sample diversity by incorporating multiple cultivars, cultivation conditions, and production areas, as well as collecting naturally bruised samples under real logistics environments. Furthermore, the relationship between bruise energy gradients and spectral responses will be systematically investigated to enhance model robustness and facilitate practical deployment in online sorting systems and intelligent logistics applications.

## CRediT authorship contribution statement

**Miaomiao Du:** Writing – original draft, Software, Methodology, Investigation, Data curation. **Jingyuan Zhao:** Writing – original draft, Software, Methodology, Investigation, Data curation. **Mengyao Wang:** Formal analysis. **Ye Song:** Formal analysis. **Xiaodong Zheng:** Formal analysis. **Kang Tu:** Supervision, Resources. **Weijie Lan:** Writing – review & editing, Supervision. **Qi Wang:** Writing – review & editing, Supervision. **Leiqing Pan:** Writing – review & editing, Supervision, Resources, Project administration, Conceptualization.

## Declaration of generative AI and AI-assisted technologies in the writing process

During the preparation of this work the author(s) used ChatGPT in order to improve the readability of the thesis. After using this tool/service, the author(s) reviewed and edited the content as needed and take(s) full responsibility for the content of the publication.

## Declaration of competing interest

The authors declare that they have no known competing financial interests or personal relationships that could have appeared to influence the work reported in this paper.

## Data Availability

Data will be made available on request.
