# Supplementary material for: Pixel-level bruise area quantification in strawberries using dual-band hyperspectral imaging and Efficient1DNet: Toward real-time quality monitoring
Source: Food Chem X. 2026 Apr 17;35:103881. doi: 10.1016/j.fochx.2026.103881 (PMC13112394; doi:10.1016/j.fochx.2026.103881)
Supplement: Supplementary file 1 — Supplementary material [file mmc1.docx]

**Supplementary Materials**

**Caption:**

**Fig. S1** The changes in *b^*^* (yellowness) values of 'Hongyan' strawberries with different bruising levels during 2 days of storage.

**Fig. S2** Original Vis-NIR (A-D) and SWIR (E-H) reflectance curves of strawberries with different bruising levels at 0 h, 12 h, 24 h, and 48 h.

**Fig. S3** Average Vis-NIR (A-D) and SWIR (E-H) reflectance curves of strawberries with different bruising levels at 0 h, 12 h, 24 h, and 48 h.

**Fig. S4** Average Vis-NIR and SWIR reflectance curves of strawberries at 0 h, 12 h, 24 h, and 48 h under the same bruising severity ((A, B) Intact; (C, D) Bruised I; (E, F) Bruised II).

**Fig. S5** Validation set confusion matrices for (A-C) storage-time classification with identical bruise levels, and (D) classification models across all storage intervals (0-48 h) using the optimal Vis-NIR-Efficient1DNet model.

**Fig. S6** Performance of the optimal Vis-NIR-Efficient1DNet model: accuracy (A,C,E,G) and loss curves (B,D,F,H) for different bruising levels at 0 h, 12 h, 24 h, and 48 h; Accuracy (I,K,M) and loss (J,L,N) curves for storage-time classification with identical bruise levels; Accuracy and loss curves (O,P) for classification models across all storage intervals.

**Table S1** Storage time classification models of strawberries with identical bruise levels based on Vis-NIR-HSI.

**Table S2** Storage time classification models of strawberries with identical bruise levels based on SWIR-HSI.

**Table S3** Description of the 1DCNN structure.

**Table S4** Description of the LSTM structure.

**Table S5** Description of the Efficient1DNet structure.


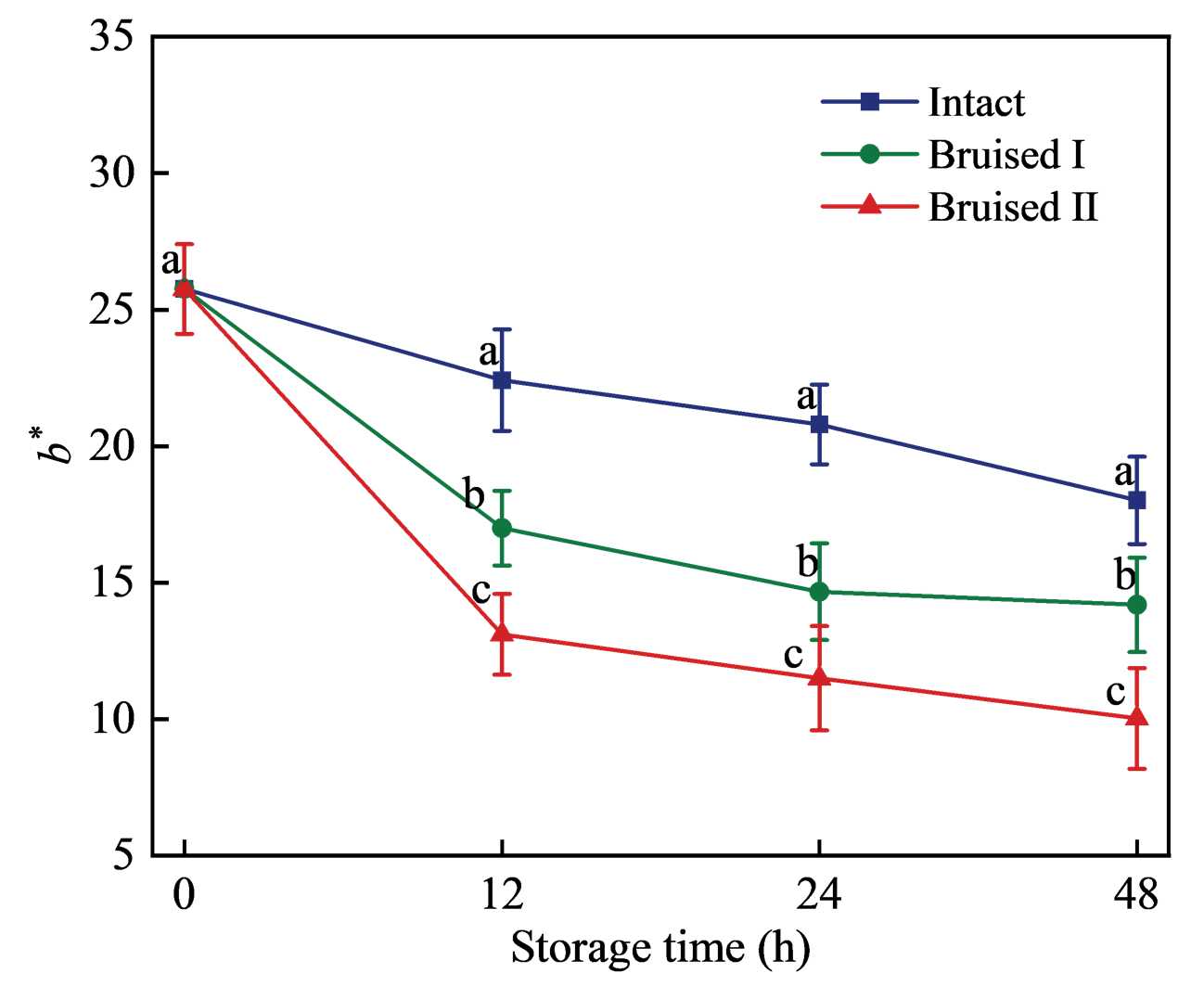


**Fig. S1.**


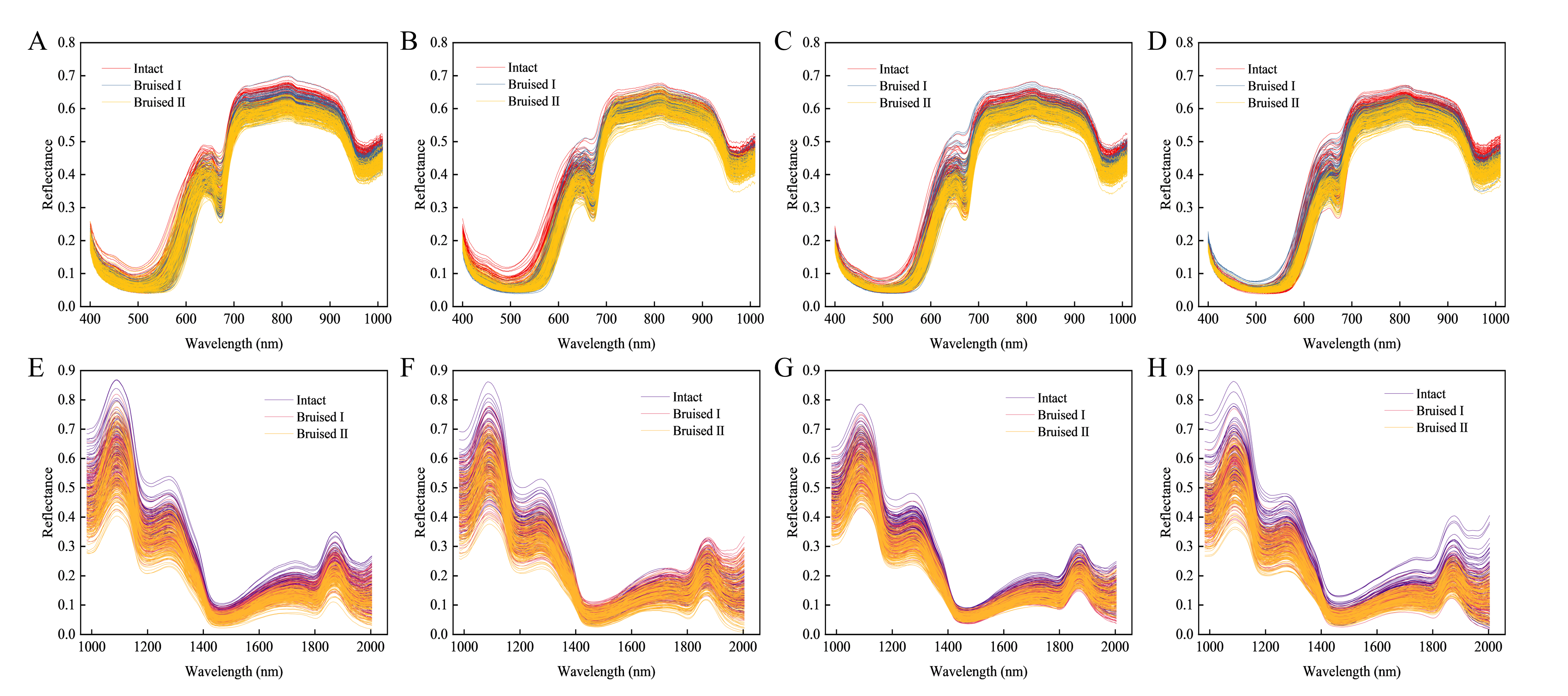


**Fig. S2.**


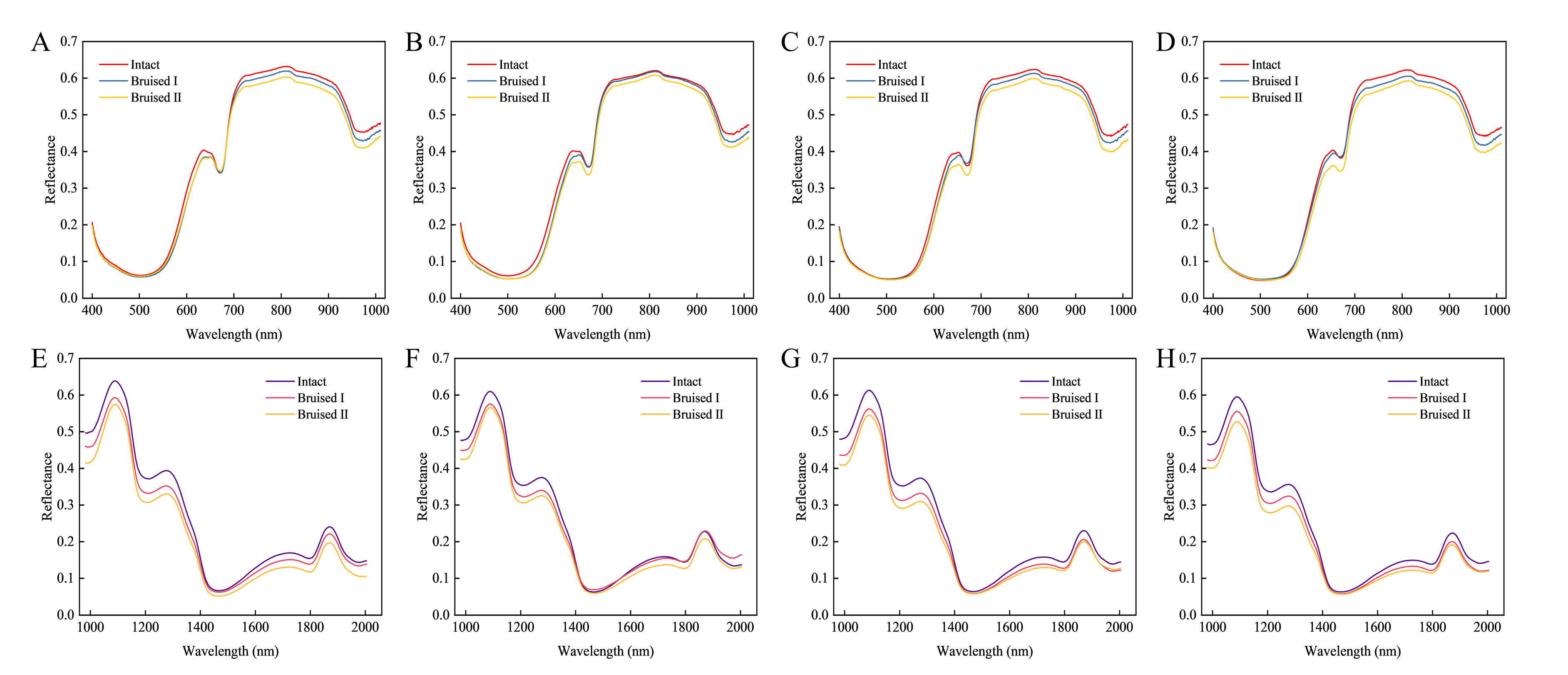


**Fig. S3.**


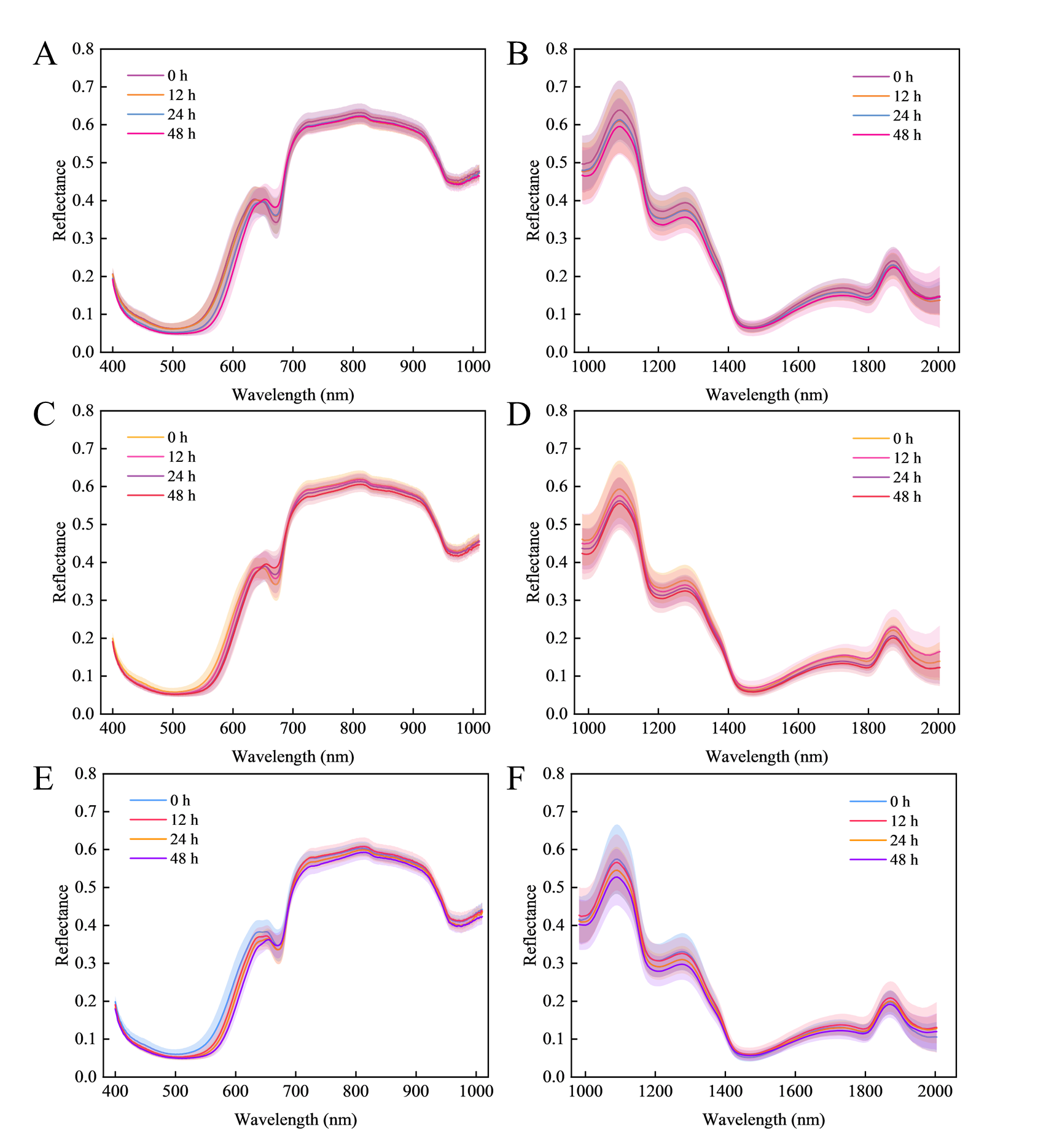


**Fig. S4.**


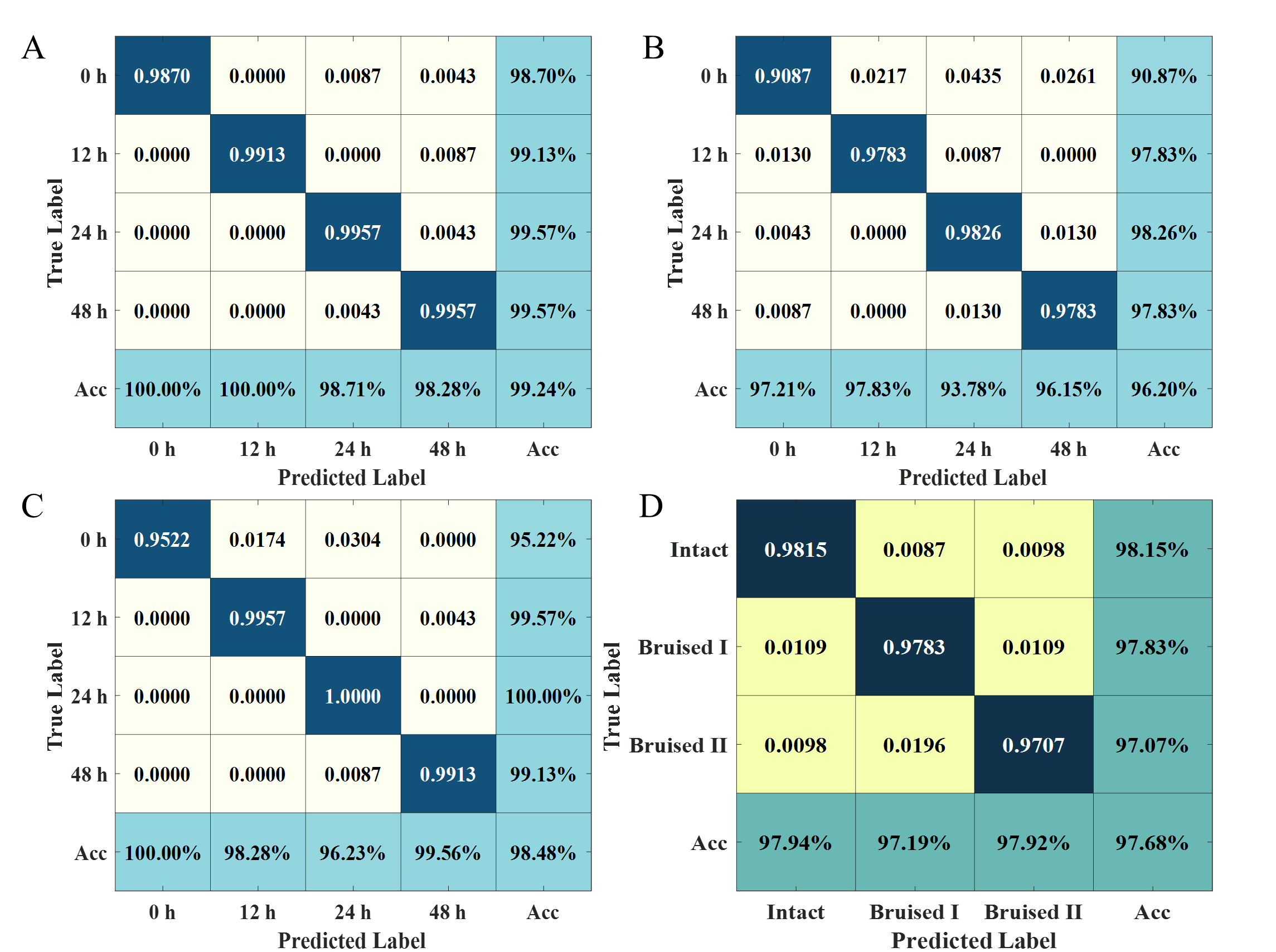


**Fig. S5.**


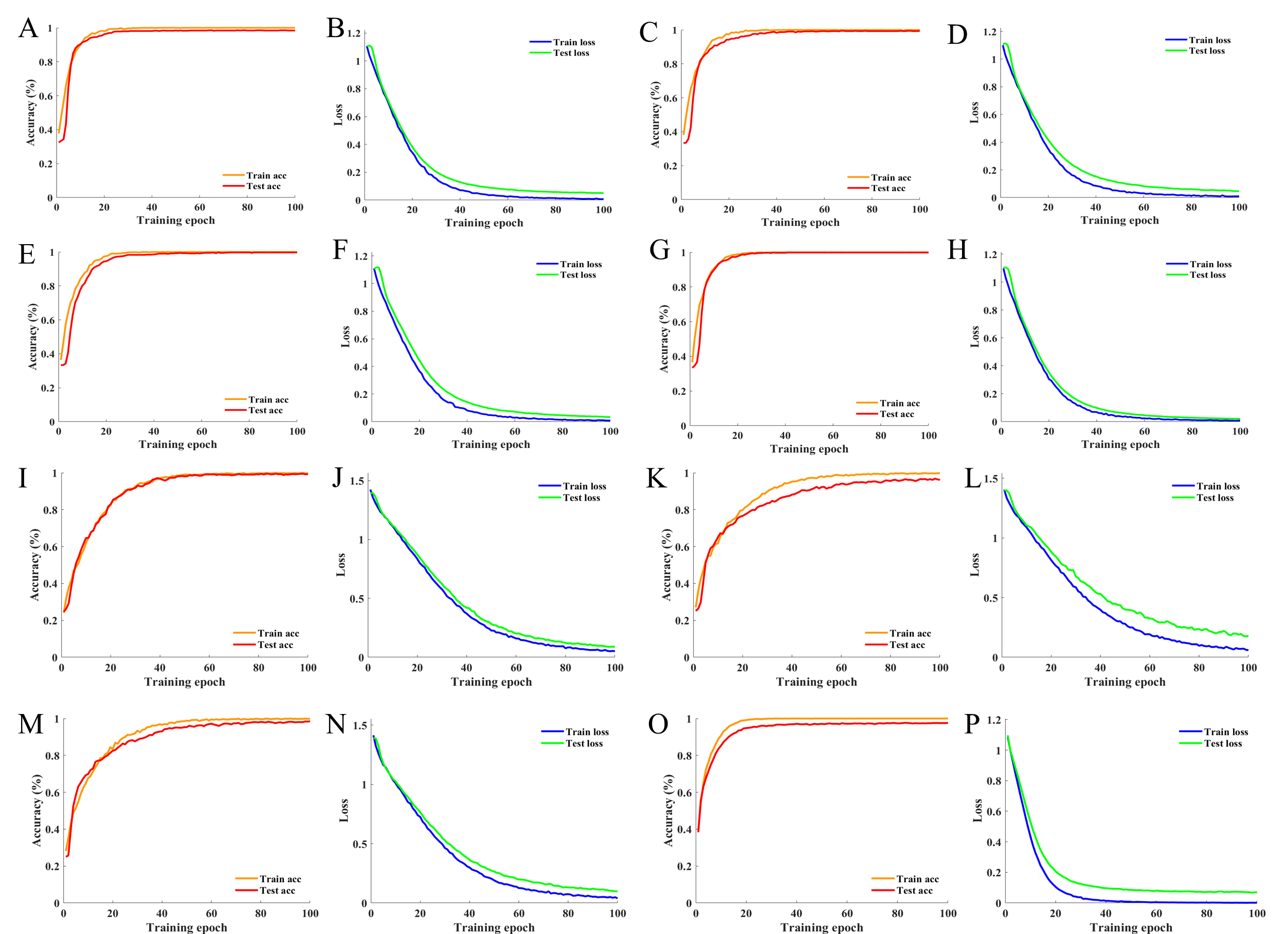


**Fig. S6.**

**Table S1** Storage time classification models of strawberries with identical bruise levels based on Vis-NIR-HSI.

| Models | Degree of Bruising | **Calibration set (%)** | | | | |  | **Validation set (%)** | | | | |
| --- | --- | --- | --- | --- | --- | --- | --- | --- | --- | --- | --- | --- |
|  |  | Storage time (h) | | | | | | | | | | |
|  |  | 0 | 12 | 24 | 48 | Overall |  | 0 | 12 | 24 | 48 | Overall |
| SVM | Intact | 97.75 | 97.75 | 98.88 | 98.88 | 98.31 |  | 93.48 | 100.00 | 97.83 | 100.00 | 97.83 |
|  | Bruised Ⅰ | 92.13 | 97.75 | 93.26 | 96.63 | 94.94 |  | 84.78 | 100.00 | 93.48 | 95.65 | 93.48 |
|  | Bruised Ⅱ | 89.89 | 88.76 | 97.75 | 98.88 | 93.82 |  | 89.13 | 86.96 | 95.65 | 100.00 | 92.93 |
| PLS-DA | Intact | 92.13 | 87.64 | 98.88 | 95.51 | 93.54 |  | 91.30 | 86.96 | 93.48 | 89.13 | 90.22 |
|  | Bruised Ⅰ | 86.52 | 96.63 | 96.63 | 98.88 | 94.66 |  | 82.61 | 97.83 | 95.65 | 97.83 | 93.48 |
|  | Bruised Ⅱ | 79.78 | 92.13 | 100.00 | 98.88 | 92.70 |  | 71.74 | 95.65 | 100.00 | 100.00 | 91.85 |
| 1DCNN | Intact | 83.37 | 86.52 | 85.39 | 83.37 | 84.66 |  | 80.04 | 84.65 | 82.61 | 82.71 | 82.50 |
|  | Bruised Ⅰ | 86.18 | 87.87 | 88.76 | 92.13 | 88.74 |  | 84.78 | 85.43 | 86.13 | 91.09 | 86.86 |
|  | Bruised Ⅱ | 92.02 | 87.19 | 91.01 | 89.44 | 89.92 |  | 86.96 | 84.61 | 87.83 | 88.33 | 86.93 |
| LSTM | Intact | 100.00 | 99.55 | 100.00 | 100.00 | 99.89 |  | 93.04 | 98.70 | 96.52 | 95.22 | 95.87 |
|  | Bruised Ⅰ | 95.81 | 96.63 | 95.96 | 98.88 | 96.82 |  | 92.13 | 93.87 | 94.00 | 95.43 | 93.86 |
|  | Bruised Ⅱ | 97.53 | 96.63 | 99.10 | 98.88 | 98.03 |  | 96.96 | 94.83 | 96.57 | 95.65 | 96.00 |
| **Efficient1DNet** | **Intact** | **100.00** | **99.78** | **100.00** | **100.00** | **99.94** |  | **98.70** | **99.13** | **99.57** | **99.57** | **99.24** |
|  | **Bruised Ⅰ** | **100.00** | **100.00** | **99.78** | **100.00** | **99.94** |  | **90.87** | **97.83** | **98.26** | **97.83** | **96.20** |
|  | **Bruised Ⅱ** | **99.78** | **100.00** | **100.00** | **100.00** | **99.94** |  | **95.22** | **99.57** | **100.00** | **99.13** | **98.48** |

**Table S2** Storage time classification models of strawberries with identical bruise levels based on SWIR-HSI.

| Models | Degree of Bruising | **Calibration set (%)** | | | | |  | **Validation set (%)** | | | | |
| --- | --- | --- | --- | --- | --- | --- | --- | --- | --- | --- | --- | --- |
|  |  | Storage time (h) | | | | | | | | | | |
|  |  | 0 | 12 | 24 | 48 | Overall |  | 0 | 12 | 24 | 48 | Overall |
| SVM | Intact | 86.52 | 93.26 | 95.51 | 93.26 | 92.13 |  | 84.78 | 93.48 | 91.30 | 91.30 | 90.22 |
|  | Bruised Ⅰ | 88.76 | 86.52 | 97.75 | 98.88 | 92.98 |  | 84.78 | 86.96 | 95.65 | 97.83 | 91.30 |
|  | Bruised Ⅱ | 91.01 | 86.52 | 95.51 | 95.51 | 92.13 |  | 86.96 | 73.91 | 95.65 | 97.83 | 88.59 |
| PLS-DA | Intact | 89.89 | 95.51 | 94.38 | 95.51 | 93.82 |  | 86.96 | 91.30 | 93.48 | 91.30 | 90.76 |
|  | Bruised Ⅰ | 93.26 | 89.89 | 97.75 | 96.63 | 94.38 |  | 89.13 | 82.61 | 93.48 | 91.30 | 89.13 |
|  | Bruised Ⅱ | 95.51 | 92.13 | 97.75 | 96.63 | 95.51 |  | 82.61 | 91.30 | 93.48 | 95.65 | 90.76 |
| 1DCNN | Intact | 80.11 | 89.10 | 88.09 | 86.74 | 86.01 |  | 78.17 | 86.78 | 85.87 | 85.39 | 84.05 |
|  | Bruised Ⅰ | 85.73 | 82.47 | 90.34 | 90.34 | 87.22 |  | 82.13 | 81.48 | 88.70 | 86.74 | 84.76 |
|  | Bruised Ⅱ | 89.89 | 89.66 | 88.99 | 84.49 | 88.26 |  | 86.43 | 87.61 | 85.87 | 83.13 | 85.76 |
| LSTM | Intact | 84.04 | 86.82 | 88.36 | 85.84 | 86.27 |  | 83.91 | 80.43 | 84.61 | 82.61 | 82.89 |
|  | Bruised Ⅰ | 89.44 | 85.39 | 86.97 | 84.61 | 86.60 |  | 85.22 | 83.22 | 84.57 | 82.83 | 83.96 |
|  | Bruised Ⅱ | 91.46 | 92.47 | 88.88 | 86.97 | 89.95 |  | 90.00 | 88.26 | 85.22 | 84.78 | 87.07 |
| **Efficient1DNet** | **Intact** | **90.74** | **95.06** | **96.11** | **94.69** | **94.15** |  | **88.35** | **93.65** | **94.09** | **92.70** | **92.19** |
|  | **Bruised Ⅰ** | **93.03** | **92.70** | **97.53** | **96.26** | **94.88** |  | **91.70** | **90.61** | **95.04** | **93.26** | **92.65** |
|  | **Bruised Ⅱ** | **95.33** | **96.45** | **95.28** | **97.79** | **96.21** |  | **92.09** | **94.52** | **93.04** | **95.87** | **93.88** |

**Table** **S3** Description of the 1DCNN structure.

| **1DCNN** |
| --- |
| Input |
| Convolution (Relu) 16@3 × 3  Convolution (Relu) 32@3 × 3 |
| Batch normalization |
| Max-pooling 1D 2 ×1 |
| Batch normalization |
| Dropout |
| Flatten |
| Fully connected |
| Dropout |
| Fully connected (Output) |

**Table S4** Description of the LSTM structure.

| **LSTM** |
| --- |
| Input |
| Convolution (Relu) 16@3 × 1 |
| Batch normalization |
| Convolution (Relu) 32@3 × 1 |
| Batch normalization  LSTM  Self-Attention  Fully Connected  Dropout |
| Fully Connected (FC2) |

**Table S5** Description of the Efficient1DNet structure.

| **Efficient1DNet** |
| --- |
| Input |
| Conv1D (16, 3×1), BN, SiLU |
| MBConv1D Block 1: 16→24, expansion=4, stride=1  MBConv1D Block 2: 24→40, expansion=4, stride=2  MBConv1D Block 3: 40→80, expansion=4, stride=2  MBConv1D Block 4: 80→120, expansion=4, stride=1  MBConv1D Block 5: 120→192, expansion=4, stride=2 |
| Adaptive Avg Pooling (output size = 1) |
| Dropout |
| Fully Connected (FC2) |
